# Drug resistance mechanisms of cancer stem-like cells and their therapeutic potential as drug targets

**DOI:** 10.20517/cdr.2019.36

**Published:** 2019-09-19

**Authors:** Takahiko Murayama, Noriko Gotoh

**Affiliations:** Division of Cancer Cell Biology, Cancer Research Institute, Kanazawa University, Kanazawa 920-1192, Japan.

**Keywords:** Cancer stem-like cell, drug resistance, epithelial-to-mesenchymal transition, hypoxia, quiescence

## Abstract

Despite of recent advances in cancer research and development of new anti-cancer drugs, tumor patients’ prognoses have not yet been improved well enough. Treatment failure of tumors is highly attributed to the drug resistance of a small population of cancer cell known as cancer stem-like cells (CSCs). CSCs also have the self-renewal activity and differentiation potency, conferring strong tumorigenicity on them. Therefore, development of CSC targeting therapy is urgently needed in order to overcome possible recurrence and metastasis by them after therapy. CSCs show some characteristic features that are not observed in other differentiated cancer cells, which give them higher resistance against conventional chemotherapy or radiotherapy. Targeting such specific features could be useful for CSC eradication. This review will summarize the recent advances in the study of CSC characteristics along with the promising therapeutic strategies targeting them.

## Introduction

**Figure 1:**
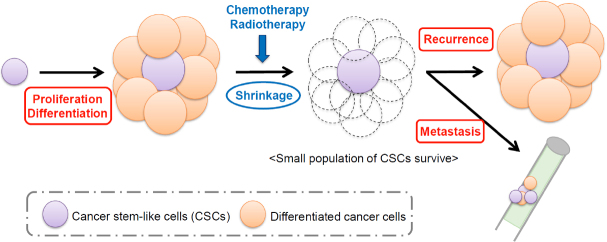
Schematic model of CSC-derived recurrence and metastasis. CSCs are thought to possess high tumorigenic potential because they have the self-renewing activity and the potency to generate differentiated cell populations. CSCs also survive after treatment with chemotherapy or radiotherapy, which leads to recurrence and metastasis, two major causes of poor patient prognosis. CSCs: cancer stem-like cells

Tumors are composed of highly heterogeneous populations, in which the existence of tumor initiating cells or cancer stem-like cells (CSCs) has been suggested. CSCs are thought to possess high tumorigenic potential because they show the normal tissue stem-like properties including the self-renewing activity and the potency to generate differentiated cell populations^[1]^. Multiple studies have also shown that the CSC population is highly resistant to conventional chemotherapeutic agents and radioactive therapies due to many kinds of properties specific to them^[2-4]^. Therefore, it is very likely that CSCs survive after treatment with conventional therapy regimens, and recurrences and metastasis, which are the major cause of poor patient prognosis, are driven by those cells, even if the number of survived cancer cells reached to an undetectable level [Figure 1].

In order to overcome the therapy resistance of CSCs, deep understanding on their biologic features is indispensable. Identification of CSC surface markers and development of functional assays including sphere forming assay, *in vivo* limiting dilution assay and aldefluor assay, have helped researchers to shed light on CSC features^[5,6]^, although there are still much to be elucidated.

In this article, we review current knowledge on the mechanisms by which CSCs show higher therapy resistance and introduce some therapeutic strategies targeting the features of CSCs.

## Representative properties of CSCs

The existence of CSCs were first indicated in 1994 by Lapidot *et al*.^[7]^. They separated patient-derived acute myeloid leukemia (AML) cells based on the expression of CD34 and CD38, which are the important markers for detection of immature cells in normal bone marrow. When transplanted into severe combined immuno-deficient mice, CD34^+^CD38^-^ subpopulation formed tumors much more efficiently than CD34^+^CD38^+^ or CD34^-^ subpopulations. Furthermore, tumors formed by CD34^+^CD38^-^ subpopulation precisely replicated many aspects of human AML. These results suggested that AML stem cells are included in CD34^+^CD38^-^ fraction. Then, in 1997, the concept of CSCs was firmly established by focusing the similarity with normal stem cells by Bonnet *et al*.^[8]^. After a decade, existence of CSCs started to be confirmed also in solid tumors including, but not limited to, breast cancer^[9]^, glioblastoma^[10]^, prostate cancer^[11]^, colorectal cancer^[12-14]^ and lung cancer^[15]^.

Although prospective markers to identify CSCs differ among tumor types, CSCs in many kinds of tumors share some important features; higher tumorigenic potential, potency to generate highly heterogeneous population of cancer cells, capacity of serial tumor propagation and higher drug resistance^[16-19]^. Self-renewing ability and multi differentiation potency are special features of normal tissue stem cells. Therefore, some researchers think that CSCs arise when tumorigenic mutations accumulate in normal stem cells. However, others think that CSCs are produced by differentiated cancer cells which undergo dedifferentiation into stem-like state, because data suggesting that cancer cells have plasticity are also accumulating^[20]^. Hence, the origin of CSCs still remains to be elucidated.

In CSCs of many types of tumors, stemness related signaling pathways including Wnt, Hedgehog and Notch are often activated^[21]^. Wnt signaling has been reported to be involved in the embryogenesis and organ development by regulating self-renewal and differentiation of normal stem cells^[22]^. When Wnt binds to frizzled receptor, it downregulates the function of AXIN/glycogen synthase kinase-3 (GSK-3)/adenomatous polyposis coli (APC) complex, leading to stabilization of β-catenin. β-catenin binds to TERT promoter region and directly enhance TERT expression, leading to maintain long telomeres, which is one of the major hallmarks of CSCs^[23]^.

Hedgehog signaling is also important for the embryogenesis and the repair of normal tissues^[24]^. When Hedgehog ligands including Sonic Hedgehog, Indian Hedgehog or Desert Hedgehog, bind to the Smoothened receptors, GLI transcription factor is activated by the receptor. The activated GLI localizes into nucleus and induce expression of genes involved in cell survival and proliferation^[25]^. Activation of Hedgehog signaling could be the cause of epithelial-to-mesenchymal transition (EMT), a representative characteristic of CSCs^[26,27]^.

Notch signaling is an evolutionarily highly conserved signaling mechanism, which is critical for cell proliferation, differentiation, organ development and homeostasis^[28]^. Unlike Wnt or Hedgehog signaling, Notch signaling occurs through cell-cell communication. When a transmembrane ligand on one cell binds to a transmembrane receptor on a neighboring cell, the receptor is cleaved and interact with nuclear factors to regulate gene expression. In mammals, there are four types of receptors, Notches 1-4, and five types of ligands, delta-like ligand 1 (DLL1), DLL3, DLL4, Jagged-1 and Jagged-2^[29]^. Although dependency on each type of ligand and receptor is varied among different tumor types, activation of Notch signaling has been interrelated with cancer stemness^[30-33]^. Therefore, Notch signaling inhibitors have been developed^[34-36]^, and the efficacy of combinational therapy with conventional anti-cancer drugs is investigated.

## Therapy resistance of CSCs

Drug resistance of cancer cells is the main reason of poor prognosis of tumor patients. If cancer cells can survive after therapy, they give rise to relapse and metastasis, which are the almost all the causes of cancer related death^[37]^.

As identifying CSCs correctly is still difficult, the relationship between CSCs and drug resistance is not completely clear. However, accumulating evidences have suggested that CSCs possess multi-drug resistance and contribute to incomplete therapeutic responses of tumors. One example indicating CSCs contribution to drug resistance is that when tumors were treated with conventional anti-cancer drugs such as cisplatin, small number of cancer cells survived and the proportion of cells showing the CSC properties significantly increased^[38,39]^. Another example is that patient prognosis is much worse when cancer cells in tumor strongly express CSC markers like CD133^[40-44]^. Furthermore, CSCs isolated from clinical breast tumor samples were reported to show strong resistance to various chemotherapeutic drugs^[42]^.

**Figure 2:**
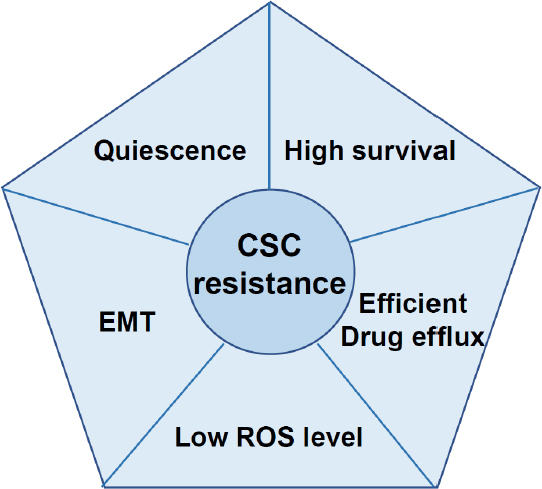
Characteristic of CSCs contributing to higher drug resistance. Higher therapeutic resistance of CSCs is maintained by many features including quiescence, epithelial-to-mesenchymal transition, low ROS level, efficient drug efflux ability and upregulated survival signaling. CSCs: cancer stem-like cells

Higher resistance of CSCs is observed not only for chemotherapy but also for radiotherapy. Bao *et al*.^[3]^ reported that once patients were irradiated in therapeutic regimen, survived glioblastoma cells became more resistant to irradiation, suggesting that CSCs were not eradicated and started to self-renew after therapy. Very recently, Carruthers *et al*.^[45]^ shed light on how glioblastoma CSCs possess higher resistance to radiotherapy. They found that DNA damage response is constitutively activated in CSCs, because of higher level of replicative stress generated in those cells. Also, based on the findings, they indicated that inhibition of DNA damage checkpoint pathway would be the promising strategy to target CSCs. Not limited to enhanced DNA damage response, CSCs have many different mechanisms to exhibit their higher therapy resistance as described below [Figure 2].

## Mechanisms of CSCs’ therapy resistance and their potential as therapeutic targets

Some features of CSCs have been linked to the higher drug resistance of the cells. Most of the features are shared with normal tissue stem cells, but some are limited to CSCs. In order to develop efficient and non-harmful therapeutic strategies, we have to identify and target therapy resistant mechanisms that are highly specific to CSCs.

### Quiescence

CSCs are thought to be in a dormant state (or referred to as G0 phase) especially after tumors are formed. Therefore, it is difficult to eliminate them with conventional anti-cancer drugs or radioactive rays which target actively proliferating cells^[46]^. Quiescent cells also have long time to repair DNA damage induced by chemotherapeutic drugs or radioactive rays. In fact, a variety of studies have shown that CSCs are highly resistant to DNA damages due to its ability to repair them^[1,47]^. In addition to time for repair, CSCs show enhanced DNA repair activity, which also contribute to its higher resistance^[45]^.

To overcome therapy resistance derived from quiescence of CSCs, researchers are trying to develop strategies to wake them up^[48]^. Takeishi *et al*.^[49]^ made it clear by using a mouse model that genetic ablation of F-box protein Fbxw7 made leukemia CSCs enter the cell cycle. Therefore, combinational therapy of Fbxw7 depletion and anti-cancer drug imatinib attenuated leukemia development not only during treatment but also after discontinuation of medication. This result strongly suggests that CSCs can be eliminated if they are not in a dormant state. In addition, Prost *et al*.^[50]^ reported that agonists of peroxisome proliferator-activated receptor-γ also inhibits leukemia CSCs to stay in a non-proliferating state by decreasing expressions of hypoxia-inducible factors 2α (HIF2α) and CITED26, which are key modulators of the quiescence and stemness. Their results also show that waking up strategy is really promising to eradicate CSC pool.

### Epithelial-to-mesenchymal transition

EMT and its counterpart, mesenchymal-to epithelial transition are the essential processes of embryonic morphogenesis^[51]^. Not limited to normal tissue stem cells, EMT is also important features of CSCs. Actually, when EMT programme was induced in human mammary epithelial cells, the cells obtained the ability to form mammary tumors in mice^[52]^. They also showed that cells that undergone EMT acquired a CD44^high/^CD24^low^ expression pattern, formed mammospheres and made soft agar colonies efficiently. As these features are often linked to breast CSCs, the EMT programme may contribute to maintain the CSC features. In addition, loss of epithelial features has been strongly linked to the ability of metastasis and patient poor prognosis^[53,54]^.

Cells which undergo EMT programme sometimes get into a dormant state, which is one mechanism in which EMT contributes to obtaining therapy resistance. However, this resistance can be attributed to various mechanisms. Farmer *et al*.^[55]^ reported that upregulation of stroma cell-related gene sets, which can be induced by EMT, correlated with higher drug resistance of breast cancer. Based on the findings, they suggested anti-stromal agents may offer chances to overcome drug resistance. Other research groups are now focusing on the way to inhibit EMT. EMT can be prevented by interrupting the signaling pathways which is critical to EMT induction. TGFβ signaling is the most characterized pathway which can induce EMT programme, therefore, inhibitors targeting this pathway has been already developed and some of them are being tested in clinical trials^[56]^. On the other hand, Jiao *et al*.^[57]^ investigated the effectiveness of curcumin, a hepatocyte growth factor (HGF) inhibitor, based on the notion that HGF signaling also contributes to EMT induction. They reported that curcumin treatment induced expression of epithelial marker E-cadherin and inhibited tumor progression, indicating the possibility that we can inhibit EMT programme by targeting HGF signaling.

### Hypoxia and low reactive oxygen species (ROS) level

Hypoxia has been reported to maintain CSCs in an undifferentiated state^[58,59]^. Jogi *et al*.^[58]^, showed that in cells exposed to hypoxia, genes involved in stemness like Notch were upregulated while differentiated markers were downregulated . The main factors which correlate hypoxia and cancer stemness are HIFs. Human HIF family is composed of HIF1α, HIF1β, HIF2α, HIF2β, HIF3α and HIF3β. HIFα subunits undergo proteasome-dependent degradation in normal oxygen conditions (normoxia) and only in the hypoxic conditions, they form complex with HIFβ subunits and become stabilized^[60]^. After HIF complex is formed, it moves into the nucleus and binds to the *Hypoxia Response Element* gene promoters to activate HIF-regulated genes.

Among the HIF family members, HIF1α and HIF2α are regarded to be important for maintaining cancer stemness. Although the DNA binding sites of the two proteins closely resemble, some of the target genes are unique to either HIF1α or HIF2α. In general, HIF1α activates survival genes in low oxygen conditions and HIF2α binds to the promoter of stemness related genes like Oct-4 and Nanog^[59,61]^. Heddleston *et al*.^[59]^, directly showed that forced expression of non-degradable HIF2α in glioma cells induced those cancer stem cell markers expression.

Researchers have investigated the effectiveness of HIF inhibitors. In order to obtain the proof of concept, Burkitt *et al*.^[62]^ compared the effect of sunitinib, a small molecule multitargeted receptor tyrosine kinase inhibitor, on colon cancer with or without disruption of HIF1α and/or HIF2α genes. They found that cells depleted HIF1α or HIF2α, or both of them showed improved therapeutic response to sunitinib and complete remission rate raised to 50% of tested mice. Based on the findings like this, some HIF1 inhibitors including BAY 87-2243, 2-Methoxyestradiol and PX-478 2HCl are currently tested in clinical trials.

In addition to direct targeting of HIFs, upstream regulator like phosphatidylinositol-3 kinase (PI3K)/Akt signaling axis can be inhibited to downregulate the function of HIF1α and HIF2α. Under hypoxic conditions, it is reported that PI3K phosphorylates Akt, which then activate gene expressions of HIF1α and HIF2α^[63,64]^. Therefore, inhibition of this signaling axis may contribute to eradicate CSCs by decreasing the function of HIF1α and HIF2α. According to reports by Desalvo *et al*.^[65]^ and Zhang *et al*.^[66]^, PI3K/Akt signaling pathway inhibitors enhanced the therapeutic efficacy of 2-deoxy-D-glucose, which is a compound that inhibits glycolysis. In addition, BEZ235, which is a dual inhibitor of PI3K and mammalian target of rapamycin (mTOR), was shown to be effective to suppress the stemness of CSCs in colon cancer by inhibiting PI3K/Akt/mTOR signaling^[67]^.

Interestingly, Liu *et al*.^[68]^ reported that E3 ubiquitin ligase Parkin, of which mutation is strongly linked to familial Parkinson’s disease, interacted with HIF1α and promoted degradation of HIF1α through ubiquitination. MDA-MB-231 breast cancer cells overexpressing Perkin showed much less ability to metastasize to lung than control MDA-MB-231 cells. Their results indicated that downregulating HIF1α function by modulating Perkin expression could hamper the CSC activity.

For both normal cells and cancer cells, accumulation of ROS causes cell death. However, it is known that in CSCs, ROS level is maintained relatively low by the enhanced expression of aldehyde dehydrogenase (ALDH)^[69]^. ALDH decreases oxidative stress, particularly caused by aldehydes^[70]^. As high expression level of ALDH correlates with high tumorigenicity and resistance to chemotherapeutic drugs, it is widely used as a CSC marker^[71-73]^. Also, according to reports by Croker *et al*.^[74]^, the specific ALDH inhibitor diethylaminobenzaldehyde sensitized ALDH^high^/CD44^+^ breast cancer cells to chemotherapy and radiotherapy. ALDH, therefore, could be a potential drug target of CSCs as well as a molecular marker.

### Overexpression of ATP-binding cassette (transporter proteins

Among many types of transporter proteins, ATP-binding cassette (ABC) transporter family, with 49 family members classified in 7 gene subfamilies, plays important roles for inducing drug resistance of cancer cells^[75]^. Most ABC transporters mediate the transport of substrate proteins (including drugs) across the plasma membrane using energy obtained by ATP hydrolysis. Overexpression of these ABC transporters has been reported in some types of cancers and especially in CSCs^[76,77]^.

Several signaling pathways are reported to be involved in the expression of ABC transporters. Some members including ABCC1 and ABCC4 are positively regulated Myc, while ABCC3 is negatively regulated by it^[78]^. The stemness-related transcription factor Oct-4 also control gene expression of ABC family members^[79]^. In addition, HMGA1, which is essential for the cellular reprogramming of somatic cells to induced pluripotent stem cells, was reported to regulate ABCG2 promoter activity through HMGA1 binding sites^[80]^. Chun *et al*.^[81]^ showed that inhibition of EGFR/HER2 signaling by lapatinib suppressed expression of ABCB1 and ABCG2, leading to sensitize breast cancer tumorsphere cells to doxorubicin. Nakanishi *et al*.^[82]^ also reported that by inhibiting BCR-ABL and its downstream PI3K/Akt signaling pathway, the protein level of ABCG2 was downregulated in chronic myelogenous leukemia cell.

Because the functions of ABC transporters are involved in the clinical multi drug resistance, combination therapies of ABC transporter inhibitors and some anti-cancer drugs have been tested. The inhibitor of ABCG2 induced the drug efficacy of mitoxantrone and topotecan to lung cancer cells which overexpress ABC transporters^[83]^. Rabindran *et al*.^[84]^ checked that Fumitremorgin C reversed the sensitivities to mitoxantrone, doxorubicin, and topotecan through inhibiting the function of ABCG2. However, as normal tissue stem cells are also maintained by the upregulated expression of ABC transporters, inhibition of the transporter family will be harmful for those cells. Furthermore, brain microvessel endothelial cells, which constitute blood brain barriers, are known to be maintained by ABC transporters including ABCG2 and ABCB1^[85]^. Therefore, eliminating CSCs by the inhibition of ABC transporters might be a double-edged sword.

### Enhanced anti-apoptotic signaling and defective pro-apoptotic signaling

CSCs also depend on the signaling pathways which contribute to preventing their apoptosis. Two major stemness related signaling pathways Notch and Hedgehog, which are often highly activated in CSCs, are shown to activate anti-apoptotic signaling. Domingo-Domenech *et al*.^[86]^ found that a subpopulation that survived Docetaxel treatment overexpressed molecules in the Notch and Sonic Hedgehog signaling pathways, and when these two pathways were inhibited, the resistant subpopulation was depleted through downregulation of Akt and B-cell lymphoma 2 (Bcl-2) expression. Bcl-2 itself is also thought to be important factor for pro-survival signaling and its upregulation has been confirmed in CSCs^[87,88]^.

In addition, genes inducing cell death are often dysregulated in CSCs. One of the leading factors regulating cell death is p53. When DNA damage or abnormal cell cycle progression is monitored, p53 is stabilized and activated^[89]^. Activated p53 then binds to DNA and induces expression of several genes involved in apoptosis. In CSCs, p53 downregulation or mutation can be often observed, which lead to the inappropriate regulation of cell death^[90,91]^. Restoration of normal p53 function is, therefore, one of the promising strategies to deprive CSCs of their therapeutic resistance^[91]^.

## Treatment approaches targeting stemness related signaling

**Figure 3:**
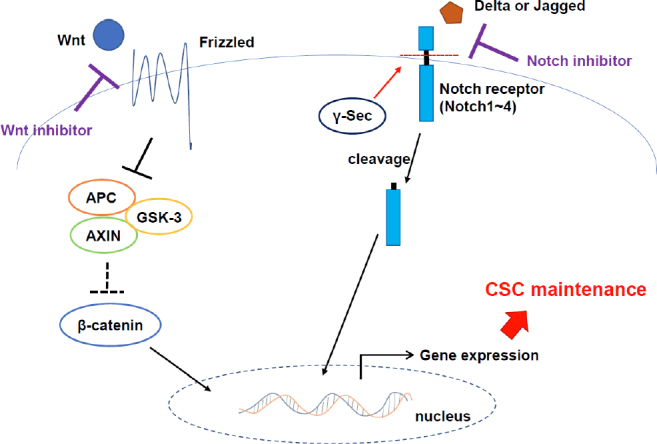
Stemness related pathways could be useful for targeting CSCs. When Wnt binds to FZD receptor, it downregulates the function of AXIN/GSK-3/APC complex, which leads to stabilization of β-catenin. Stabilized β-catenin moves into nucleus and induces the stemness related gene expressions. When a transmembrane Notch ligand (Delta or Jagged) on one cell binds to a transmembrane receptor (Notches 1-4) on a neighboring cell, the receptor is cleaved and interact with nuclear factors to regulate stemness related gene expressions. Inhibition of these pathways may contribute to CSC eradication. FZD: frizzled; GSK-3: glycogen synthase kinase-3; APC: adenomatous polyposis coli. CSCs: cancer stem-like cells

Because CSCs have potential to survive many kinds of conventional therapies, new therapeutic strategies to eradicate CSCs are now under investigation [Figure 3]. One way targeting CSCs is to inhibit the signaling pathways highly activated in those cells. Many types of Wnt signaling inhibitors, which targets ligand-receptor interaction^[92,93]^ or their downstream effectors porcupine^[94]^ or β-catenin^[95]^, have been developed, and some of them are now on the clinical trial. Notch signaling, another stemness related signaling pathway, is also a candidate target of CSCs. Curcumin, which is a natural compound produced by *Curcuma longa* plants, has been reported to inhibit this pathway^[96]^. In addition, inhibitors targeting Notch pathway has been developed and efficacy on CSC eradication of the drugs have been suggested^[97]^.

## Treatment approaches targeting other activated signaling pathways in CSCs

**Figure 4:**
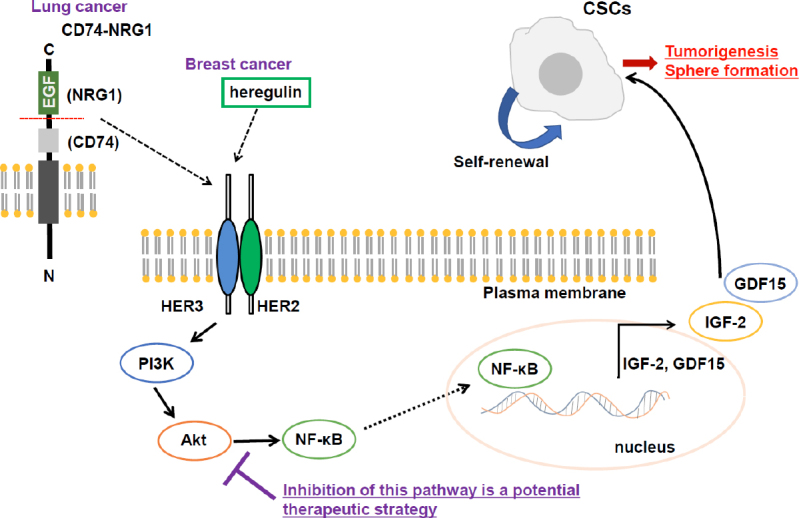
PI3K/Akt signaling pathway contributes to CSC maintenance and could be a promising target. When CD74-NRG1 fusion protein (in lung cancer) or heregulin (in breast cancer) binds to HER2/HER3 heterodimer receptor, PI3K/Akt/NF-κB signaling is activated. NF-κB induces gene expression of IGF-2 and growth differentiation factor 15 (GDF15), which enhances self-renewal ability of CSCs. Therefore, inhibition of this signaling pathway would be promising to target CSCs. IGF-2: insulin-like growth factor-2; GDF15: growth differentiation factor 15. CSCs: cancer stem-like cells

Not limited to these stemness related signaling pathways, other activated signaling could be targeted. PI3K/Akt signaling plays a crucial role in proliferation and survival of cancer cells during tumorigenesis^[98]^. We previously showed that PI3K/Akt signaling pathway activated by newly identified CD74-NRG1 fusion gene products contributed to maintenance and growth of CSCs in lung cancer^[99,100]^. By blocking this signaling pathway with PI3K inhibitor, the self-renewal activity of CSCs significantly suppressed. We have also shown that PI3K/Akt signaling pathway, which is activated by heregulin, contributes to the maintenance of CSCs in breast cancer^[101]^. Insulin-like growth factor-2 (IGF-2)^[102]^ and growth differentiation factor 15 (GDF15)^[103]^, downstream factors of heregulin/PI3K/Akt, have been shown to play important roles in CSC maintenance [Figure 4].

MAPK-ERK signaling pathway is also important for CSC maintenance. Ding *et al*.^[104]^ made it clear that CD133^+^ CSCs in liver cancer demonstrated higher resistance to TGF-β induced apoptosis than CD133^-^ non-CSCs. In their study, MAPK-ERK pathway inhibition effectively decreased CD133^+^ CSC population, thus, targeting signaling pathways highly activated in CSCs would be promising for CSC eradication.

**Figure 5:**
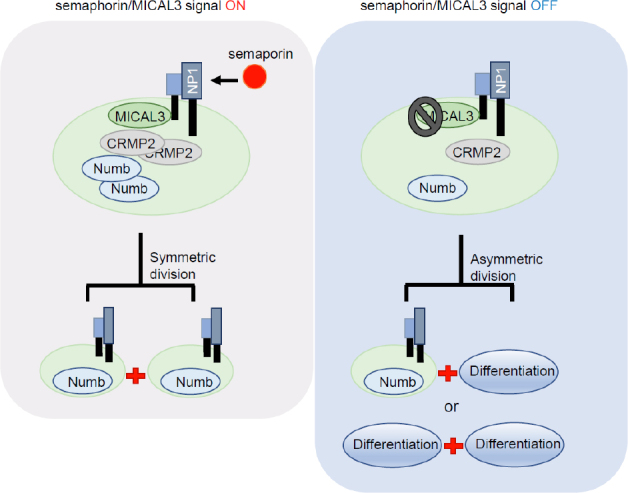
Semaphorin/MICAL3 signaling induces symmetric division of CSCs. By semaphorin binding to neuropilin 1 (NP1) receptor, MICAL3/collapsin response mediator protein 2 (CRMP2)/Numb axis is activated, leading to symmetric division and expansion of BCSCs. NP1: neuropilin 1; CRMP2: collapsin response mediator protein 2. CSCs: cancer stem-like cells

Recently, we have reported that semaphorin signaling via MICAL3/collapsin response mediator protein 2 (CRMP2)/Numb axis contributes to the maintenance of breast CSCs by inducing symmetric cell division^[105]^. Depletion or inhibition of factors included in the semaphoring/MICAL3 signaling pathway increased asymmetric cell division of CSCs, which indicated that targeting this pathway could lead to eradication of CSCs [Figure 5].

## CSC targeting strategy focusing immune system

Furthermore, targeting CSCs by immunotherapy has recently been a hot topic^[106]^. One potential approach is to generate T-cell responses. In this strategy, CSCs isolated from patient tumors are loaded onto dendritic cells (DCs), which could be used as a cancer vaccine. Lu *et al*.^[107]^ showed the efficacy of CSC-DC vaccine by using the melanoma and squamous cell carcinoma mouse models . They enriched CSCs for vaccine production based on high expression level of ALDH, and administration of the CSC-DC vaccine significantly decreased tumor volume, ALDH^high^ CSC frequency and the probability of metastasis. Based on success of preclinical studies including this one, clinical studies of CSC-DC vaccine therapy are now ongoing.

## Conclusion

CSCs show higher resistance to conventional chemotherapies and radiotherapies by staying in a dormant state, decreasing intra-cellular ROS levels, inducing export of toxic agents out of cells, or utilizing many other mechanisms as summarized in this review. Although accumulating knowledge on CSC features may enable us to target CSCs efficiently, there are still many challenges to be overcome. First, we have to distinguish the ways that will not damage normal tissue stem cells, which may not lead to the severe side effects. Second, as killing only CSCs could not succeed in complete tumor eradication, developing efficient combinational therapies will be needed. Therefore, it will take long time before the CSC targeting therapy started to be used widely in clinical settings. Though, once such a strategy is established, much more efficient tumor therapy will come true in the future.

## Declarations

### Copyright

© The Author(s) 2019.
